# De Novo Autoimmune Hepatitis after COVID-19 Infection in an Unvaccinated Patient

**DOI:** 10.1155/2022/8409269

**Published:** 2022-12-22

**Authors:** Francisco A. Durazo, Kelly Kristbaum, James Miller, Kia Saeian, Motaz Selim, Johnny C. Hong

**Affiliations:** ^1^Division of Transplant Surgery, Department of Surgery, Medical College of Wisconsin, Milwaukee, WI, USA; ^2^Division of Gastroenterology and Hepatology, Department of Medicine, Medical College of Wisconsin, Milwaukee, WI, USA; ^3^Department of Pathology and Laboratory Medicine, Medical College of Wisconsin, Milwaukee, WI, USA; ^4^Division of Transplantation, Pennsylvania State University, College of Medicine Penn State Health Milton S. Hershey Medical Center Hershey, Pennsylvania, USA

## Abstract

Liver test abnormalities have been described during severe acute respiratory syndrome coronavirus (SARS-CoV)-2 infection causing coronavirus disease 2019. Most of them consist of elevation of the aminotransferases that resolve once the infection subsides. There are several reports of autoimmune hepatitis developing after vaccination against COVID-19 and one case of autoimmune hepatitis following COVID-19 infection. We present a patient that was not vaccinated against COVID-19 and developed resistant de novo autoimmune hepatitis following COVID-19 infection requiring aggressive immunosuppression.

## 1. Introduction

Severe acute respiratory syndrome coronavirus (SARS-CoV)-2 was first identified in December of 2019 in Wuhan City, China, and was declared a global pandemic by the World Health Organization (WHO) on March 11^th^, 2020. The disease is termed coronavirus disease 2019 (COVID-19). COVID-19 is typically characterized by fever, fatigue, dry cough, anosmia, and headache, which may evolve to respiratory failure [1]. COVID-19 infection has been associated with the development of autoimmune processes [2–5]. It seems as though the shared pathogenetic mechanisms and the clinical aspects between the hyper-inflammatory diseases and COVID-19 may suggest that SARS-CoV-2 could act as a triggering factor for the development of a rapid autoimmune and/or autoinflammatory dysregulation in genetically predisposed individuals [6]. There are several reports of autoimmune hepatitis that developed after COVID-19 vaccine [7–14] and one case report on a patient that developed autoimmune hepatitis during COVID-19 infection [15]. Molecular mimicry has been suggested as a potential mechanism for these associations [5]. We report the case of a patient, unvaccinated against COVID-19, that developed de novo autoimmune hepatitis after COVID-19 infection.

## 2. Clinical Presentation and Diagnostic Tests

A forty-nine-year-old Hispanic woman was in her usual state of health until 10/11/2020 when she presented with fever, cough, and loss of smell and taste. She saw her primary care provider (PCP) and tested positive for SARS-CoV-2 (nucleic acid amplification test per anterior nasal swab) on 10/11/20 and again on 10/21/20. She stayed home and was instructed to drink adequate liquids and to call back if shortness of breath would ensue. She recovered from her respiratory symptoms. Three weeks later she noted decreased appetite. In the beginning of December 2020, she called her primary care doctor with complaints of intermittent right upper quadrant abdominal pain, dark urine, and yellowing of the eyes. Laboratory studies revealed total bilirubin of 8.93 mg/dL, alanine aminotransferase (ALT) of 851 U/L [0–33], aspartate aminotransferase (AST) of 614 U/L [0–32], and alkaline phosphatase of 70 U/L [30–115]; therefore, she was instructed to go to the emergency department (ED).

Repeat laboratory studies in the ED revealed a total bilirubin of 10.82 mg/dL, ALT of 781 U/L, AST of 671 U/L, and alkaline phosphatase of 71 U/L. Urinalysis revealed urobilinogen of 2.0 mg/dL and a moderate amount of blood. Amylase was 67 U/L, and lipase was 101 U/L. The complete blood count showed white blood cell count of 6.1 × 10^3^, hemoglobin of 13.1 g/dL, hematocrit of 41%, and a platelet count of 258 × 10^3^. Troponin was negative. C-reactive protein was 13.68 mg/L. SARS-CoV-2 was not detected.

An ultrasound of the abdomen showed a markedly enlarged liver with normal echotexture, no intrahepatic biliary dilatation, and normal portal venous flow. The common bile duct (CBD) was dilated to eight millimeters (mm) with diffuse gallbladder wall thickening and edema but no stones.

Magnetic resonance cholangiopancreatography (MRCP) was obtained confirming diffuse gallbladder wall thickening without cholelithiasis, porta hepatis, and peripancreatic lymphadenopathy with reference to a porta hepatis lymph node measuring 4.5 by 2.5 centimeters (cm) without choledocholithiasis.

The surgical team was consulted and performed robotic assisted laparoscopic cholecystectomy with intraoperative cholangiogram and liver biopsy without complication. Liver pathology revealed variable, mild to moderate portal mononuclear cell inflammation with mixed plasma cells and eosinophils. There was no fibrosis, no iron deposition, and no bile duct injury. Further laboratory studies revealed non-reactive hepatitis A, B, and C serologies. She recovered favorably from the surgery and was discharged home.

She was referred to the Hepatology Clinic at our institution after her liver function tests did not improve post-cholecystectomy: total bilirubin increased to 11.41 mg/dL, ALT at 789 U/L, AST at 568 U/L, alkaline phosphatase at 66 U/L.

In clinic, she denied any previous problems with her liver or previous hospitalizations, and the available liver tests prior to her recent hospitalization were within normal limits. Her past medical history was significant for motion sickness. In addition to the recent laparoscopic cholecystectomy, her past surgical history was significant for a tonsillectomy, removal of a benign neck lymph node, and two benign breast mass excisions four years apart. Family history was negative for any autoimmune disorders or liver disease. She denied any herbal supplements or over-the-counter medications. She did report taking dextromethorphan/guaifenesin when experiencing flu-like symptoms after being diagnosed with COVID-19 four months prior. She reported not feeling well ever since being diagnosed with COVID-19.

She denied any recent travel, sick contacts, and blood transfusions. She had prior tattoos but none recently.

On physical examination, her vital signs were unremarkable. Physical assessment revealed an overweight female who was visibly jaundiced with no vascular angiomas and no lymphadenopathy. Lungs were clear to auscultation, and respiratory effort was normal. Heart auscultation showed a regular rate and rhythm without murmur, gallop, or rub. Her abdomen was soft, rounded, non-tender, and non-distended, with no organomegaly or masses. There was no peripheral edema. Neurological exam showed that she was alert and oriented without asterixis.

A complete serologic workup including hepatitis A IgM, hepatitis B core total and IgM, hepatitis C antibody and RNA, nuclear antibody, smooth muscle antibody, mitochondrial antibody, hepatitis E IgM, cytomegalovirus DNA, adenovirus, and Epstein–Barr DNA were all negative. Her immunoglobulin G (IgG) was 2093, alpha-1 antitrypsin was 204 mg/dL with M1M1 phenotype, and ceruloplasmin was 19 mg/dL. Iron panel showed a total iron binding capacity of 543 ug/dL, serum ferritin of 413 ng/mL, and a serum iron of 218 ug/dL. Lactate dehydrogenase (LDH) was 258 unit/L, creatinine phosphokinase (CPK) was 73 unit/L, and alpha-fetoprotein (AFP) was 7.0 ng/mL. Repeated SARS-CoV-2 (nucleic acid amplification test per anterior nasal swab) was negative.

According to the Revised Scoring System of the International Autoimmune Hepatitis Group, this patient had a score of 18 points, which is definite for the diagnosis of autoimmune hepatitis [16]. She was started on prednisone 40 milligrams (mg) daily for autoimmune hepatitis. Repeat blood work showed improvement with a total bilirubin of 3.6 mg/dL, ALT at 312 U/L, AST at 147 U/L, and alkaline phosphatase at 48 U/L. She was continued on corticosteroid therapy to which the total bilirubin responded favorably as low as 2.65 mg/dL; however, her aminotransferases remained unchanged.

She was sent for percutaneous lymph node biopsy of the enlarged porta hepatis lymph node which was unable to be completed due to position of the lymph node. Endoscopic ultrasound guided biopsy with fine needle aspiration was also attempted and aborted due to proximity of the node to the bile duct and portal vein.

She began a steroid taper that lasted four weeks. MRCP was repeated and unremarkable. Meanwhile, her total bilirubin increased to 4.51 mg/dL, ALT at 545 U/L, AST at 343 U/L, and alkaline phosphatase 63 U/L. Her prednisone was again increased to 40 mg. Her laboratory studies continued to worsen, and she complained of nausea and diarrhea. The INR slowly up trended to 2.0, and she complained of rectal bleeding. Colonoscopy was performed which revealed internal and external hemorrhoids—likely the source of rectal bleeding—as well as minimal diverticulosis.

**Figure 1:**
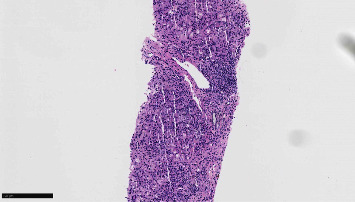
This low-power (100x) image of the second liver biopsy shows a moderate portal lymphoplasmacytic infiltrate with interface activity.

**Figure 2:**
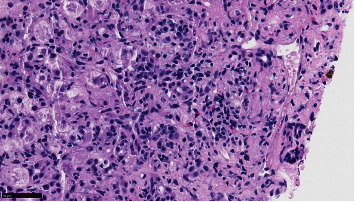
At 400x, plasma cells and eosinophils are easily identifiable as is surrounding interface activity.

**Figure 3:**
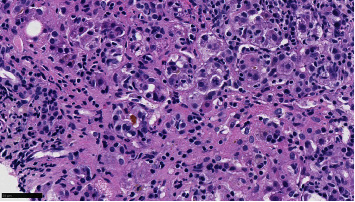
Another area shows canalicular cholestasis. Numerous background lymphocytes and plasma cells are also visible.

Vitamin K was given for three days with no improvement of PT/INR. A second liver biopsy was taken showing severe cholestatic hepatitis with an atypical lymphocytic portal and sinusoidal infiltrate, with easily identifiable plasma cells and interface activity. Viral stains were negative (Figures 1–3).

Her Model for End-Stage Liver Disease (MELD) score had increased to 25, Na-MELD of 27, therefore she was referred for liver transplant due for concern for continued decompensation. Unfortunately, during this time she started to complain of abdominal pain, presented to the ED, and was admitted under the transplant surgery service for medical management. Computerized tomography (CT) of the abdomen and pelvis demonstrated extrahepatic mild biliary ductal dilatation measuring up to 9 mm and mild perihepatic ascites and portacaval lymphadenopathy. She improved with conservative management and was discharged home. Laboratory studies at time of discharge were total bilirubin 24.8 mg/dL, ALT 284 U/L, AST 442 U/L.

She returned to the ED three days later with right upper quadrant pain. T bilirubin increased to 30 mg/dL, ALT 289 U/L, AST 425 U/L, lipase 81. Liver doppler ultrasound demonstrated normal liver morphology and hemodynamics. MRCP demonstrated mild dilatation of bile ducts with CBD measuring up to 10 mm with decreased T2 signal and diffusion restriction with associated wall thickening and enhancement. She underwent endoscopic retrograde cholangiopancreatography which showed biliary dilation without any definite stricture or filling defect. A plastic biliary stent was placed. Her total bilirubin decreased to 21.9 mg/dL, and she was discharged home when symptoms improved.

Follow-up laboratory studies revealed a total bilirubin of 23 mg/dL, ALT of 140 U/L, AST of 225 U/L, and alkaline phosphatase of 101 U/L. PT/INR increased to 2.8. She was prescribed vitamin K 10 mg daily for three days, mycophenolate 1000 mg twice daily, and prednisone 20 mg daily.

On weekly labs, her total bilirubin improved to 16, 9, 8, and 6 mg/dL, respectively, and continued to downtrend. Her INR improved to 2.4, 2.0, 1.8, and 1.9 and continued to downtrend. Her immunoglobulin G continued to remain elevated in a range of 1500–2700. Her IGG subset showed elevation in IGG1 at 2257 (Table 1).

Her CA19-9 also continued to be elevated with a high of 2149 which was attributed to cholestasis. LDH also remained elevated in the 217–246 range.

She continued with transplant evaluation despite clinical improvement on mycophenolate and prednisone therapy. Workup revealed a breast mass 10 by 13 mm on mammogram. Biopsy confirmed invasive ductal carcinoma: 90% estrogen receptor positive and 60% progesterone receptor positive. Her transplant episode was closed, and she was referred to hematology/oncology for further treatment. She was BRCA negative. She continues to require prednisone 20 mg daily and mycophenolate 750 mg twice daily as maintenance therapy for autoimmune hepatitis.

## 3. Discussion

Liver test abnormalities have been identified as one of a growing list of findings in the spectrum of non-pulmonary manifestations described in COVID-19 infection. Different studies on COVID-19 have shown an incidence of liver injury ranging from 14.8% to 53%, mainly abnormal aminotransferases [17–21]. Currently, studies on the mechanisms of SARS-CoV-2-related liver injury are limited. It has been shown that SARS-CoV-2 uses ACE2 as its entry receptor. Both liver cells and bile duct cells express ACE2 [22]. However, the ACE2 expression of the bile duct cells is much higher than that of liver cells. Another study reported that the liver injury observed in COVID-19 patients might be caused by antivirals (lopinavir/litonavir) used for the treatment of SARS-CoV-2 infection [23].

COVID-19 infection has also been associated with the development of autoimmune processes [2–5]. Severe cases of COVID-19 infection are characterized by an autoinflammatory dysregulation that contributes to tissue damage [5]. The shared pathogenetic mechanisms and the clinical aspects between the hyper-inflammatory diseases and COVID-19 may suggest that SARS-CoV-2 could act as a triggering factor for the development of a rapid autoimmune and/or autoinflammatory dysregulation in genetically predisposed individuals [6]. There are several reports of autoimmune hepatitis that developed after COVID-19 vaccine [7–14] and one case report on a patient that developed autoimmune hepatitis during COVID-19 infection [15].

We report a case of a forty-nine-year-old unvaccinated female patient that developed new-onset autoimmune hepatitis after COVID-19 infection. She never recovered fully from her initial infection and complained of decreased appetite three weeks later and presented with jaundice approximately seven weeks later. She underwent a cholecystectomy with worsening of her liver tests and an intraoperative biopsy that showed portal mononuclear cell inflammation with mixed plasma cells and eosinophils and mild macrovesicular steatosis. Her serologic workup for viral infections, autoimmune, and metabolic diseases was non-contributory. She was not taking any hepatotoxic medications or supplements. She was started on prednisone, as a therapeutic trial for autoimmune hepatitis, without improvement of her liver tests. Prednisone was tapered. Her liver tests continued to worsen, and another therapeutic trial was attempted without much success. Due to her deterioration (total bilirubin 30 mg/dL, PT INR 2.8), she was evaluated for a liver transplant. She was then started on prednisone and mycophenolate. Her liver tests finally started to improve. Attempts were made to taper down prednisone but were unsuccessful. She still remains on dual therapy with prednisone and mycophenolate.

We suspect that COVID-19 infection may have triggered her autoimmune hepatitis. She had no history of liver disease in the past, and there was a temporal relation between COVID-19 infection and the onset of her symptoms. Her liver biopsy showed no fibrosis, suggesting absence of previous disease. She was unvaccinated against COVID-19.

The eventual dramatic response to prednisone and mycophenolate also supports the diagnosis of autoimmune hepatitis. In addition, the necessity of dual therapy to control the aminotransferases points toward a resistant autoimmune hepatitis. Further attempts at tapering the dose of prednisone have been futile.

## 4. Conclusion

We report a case of new-onset autoimmune hepatitis after COVID-19 infection in an unvaccinated patient. She presented three weeks after her diagnosis of COVID-19 infection with an aggressive steroid-resistant autoimmune hepatitis requiring prednisone and mycophenolate as dual maintenance therapy to control and prevent further progression of her disease.

## Figures and Tables

**Table 1:** Evolution of liver tests after COVID-19 infection.

Date	ALT	Bilirubin	IgG	INR	Alt/10	Alt/100
2/19/2021	851	8.9	2000	1.2	0.851	8.51
2/23/2021	469	6.7	2000	1.2	0.469	4.69
3/3/2021	789	11	2093	1.1	0.789	7.89
3/5/2021	781	11	2093	1.2	0.781	7.81
3/11/2021	736	9.4	1798	1.2	0.736	7.36
3/22/2021	312	3.6	1502	1.1	0.312	3.12
4/1/2021	637	2.6	1668	1.1	0.637	6.37
4/16/2021	693	4	1668	1.1	0.693	6.93
4/29/2021	538	3.7	1862	1	0.538	5.38
5/21/2021	545	4.5	1855	1.3	0.545	5.45
6/17/2021	552	17	1850	1.4	0.552	5.52
6/25/2021	496	17	1970	1.4	0.496	4.96
7/13/2021	393	22	1964	1.8	0.393	3.93
7/19/2021	262	20	2354	2.1	0.262	2.62
7/24/2021	289	31	2354	2.3	0.289	2.89
8/12/2021	129	16	2557	2.4	0.129	1.29
8/23/2021	114	9.4	2557	1.8	0.114	1.14
9/3/2021	81	6.6	1885	1.7	0.081	0.81
9/18/2021	60	4	1885	1.4	0.06	0.6
10/8/2021	56	2.2	1565	1.3	0.056	0.56
11/19/2021	42	0.8	1565	1.2	0.042	0.42
12/20/2021	38	1	1560	1.2	0.038	0.38
1/28/2022	128	1.1	1560	1.4	0.128	1.28
2/15/2022	84	1	1502	1.3	0.084	0.84
3/24/2022	45	0.6	1502	1.2	0.045	0.45
4/21/2022	30	0.6	1502	1.1	0.03	0.3

## Data Availability

The data are available from the corresponding author upon request.
